# Uterine leiomyoma with tubules

**DOI:** 10.1186/1477-7800-5-15

**Published:** 2008-06-09

**Authors:** Teresa Pusiol, Anna Maria Parolari, Francesco Piscioli

**Affiliations:** 1 Institute of Anatomic Pathology, "S.Maria del Carmine" Hospital Rovereto, Rovereto (TN), Italy; 2 Division of Gynecology and Obstetrics, New Hospital, Arco (TN), Italy

## Abstract

We report two cases of "uterine leiomyoma with tubules" as a new pathological entity. Since these are biphasic neoplasms (composed by epithelial and mesenchimal elements), the differential diagnosis is between mixed mullerian tumors and uterine tumors resembling ovarian sex cord tumors (UTROSCTs). In the differential diagnosis, the mixed mullerian tumors are easily excluded because of histological and immunohistochemical features. UTROSCTs are similar to the lesions we reported, and the differential diagnosis requires positivity for some immunohistochemical markers as inhibin, CD99, calretinin, Melan-A. Our conclusions are that to perform a diagnosis of UTROSCT at least two immunohistochemical marker have to be expressed; in the present case they didn't, so we call the lesion "leiomyoma with tubules".

## Background

The presence of cellular structures with epithelial-like or sex cord-like appearance embedded in mesenchimal nodular circumscribed tissue is an unusual finding, and it may cause some difficulties in making the diagnosis or in classifying the lesion. In the present paper we describe the clinicopathological features of two neoplasms composed by tubules and gland-like epithelial structures and smooth muscular tissue. Histologically, the lesion simulated uterine tumors resembling ovarian sex cord tumors (UTROSCTs), but the immunophenotype was not consistent with true sex-cord differentiation. The diagnosis consequently had been only descriptive: "leiomyoma with tubules".

## Case presentation

### Case 1

In July 1997 a 55-years-old postmenopausal woman presented with recurrent vaginal bleeding. Ultrasonography preoperative diagnosis was uterine leiomyoma. The patient underwent hysterectomy and bilateral salpingo-oophorectomy. Grossly the uterus weighed 120 g and measured 10 × 5 × 5 cm. On the cut surface the uterine wall, showed a submucosal, solid, gray nodule of 3.5 cm in diameter.

### Case 2

In April 1998 a 64-years-old postmenopausal woman presented with episodic vaginal bleeding. Ultrasonography revealed a mass located within the left wall of the uterus. A standard total abdominal hysterectomy and bilateral salpingo-oophorectomy was performed. On the cut surface an 1,9 cm maximum diameter intramural nodule was localized within the anterior wall and was focally firm and white in appearance with other softer yellowish colored areas.

Three blocks for each case was selected for immunohistochemistry. Staining was performed using the following antibodies:

CAM5.2 low-weight keratin, AE1/AE3 high-weight keratin, vimentin, desmin, caldesmon, inhibin, estrogen receptor (ER), CD99, CD10, calretinin, progesterone receptor (PgR) and Melan-A. Immunostaining was carried out using appropriate positive controls. Primary antibody was detected using a sensitive Strept-ABC technique with diaminobenzidine development. All staining were performed with an automatic immunostainer (Biogenex Optimax).

Cases were scored as negative, focally positive (<50% cells staining) or diffusely positive (> 50% cells staining)

**Figure 1:**
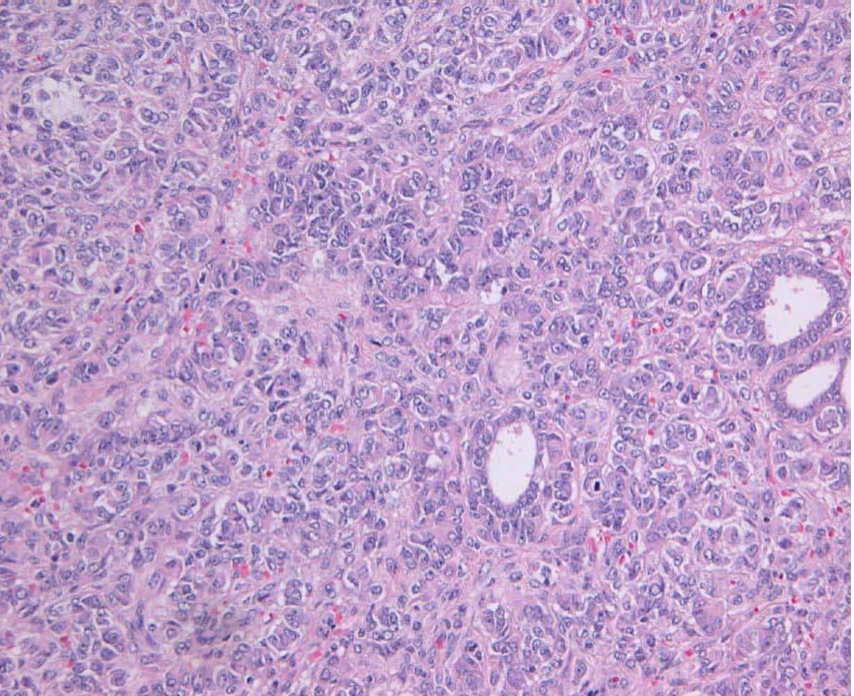
Case1. Into the mesenchymal component it is appreciable the glandular elements (Hematoxylin & Eosin, 100×).

Histologically, both neoplasms were situated in the myometrium without involvement of the endometrium. They were well circumscribed, exhibited similar features and were mainly composed of sweeping and intersecting fascicles of smooth muscle cells surrounding a diffuse proliferation of tubular and gland-like structures lined by plump cells with indistinct cytoplasm (Fig. 1). Necrosis, nuclear pleomorphism or mitoses were not observed within the tumors.

**Figure 2:**
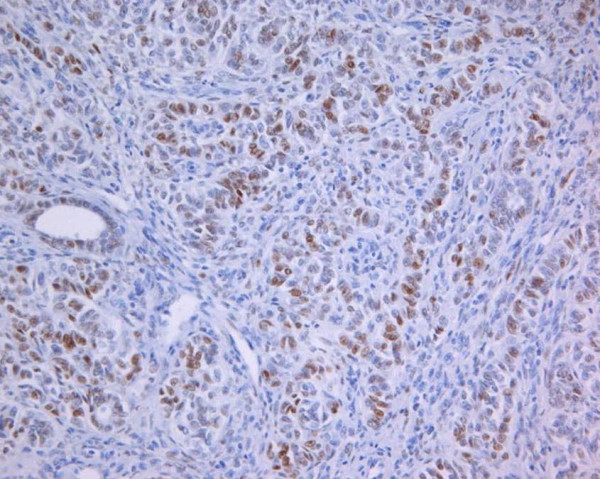
Case2. Expression of antibody anti-estrogen receptor (Estrogen receptor, 100×).

Smooth muscle elements and tubular structures were weakly immunoreactive for high and low molecular keratins (AE1/AE3 and CAM5.2) and calretinin; caldesmon and desmin were strongly positive only in the smooth muscle component. Staining for inhibin, CD99, CD10 and Melan-A were negative. Glandular structures showed nuclear cells positivity for PgR, while ER immunoreactivity was expressed into the stromal cells and glandular elements (Fig. 2). The case was sent for consultation to Prof. Hildebrandt, Prof. Hendrickson, Prof. Kempson of Laboratory of Surgical Pathology Stanford Health Services, California, USA, that performed the diagnosis of "Leiomyoma with tubules".

The diagnosis "leiomyoma with tubules was performed for both cases.

## Discussion and conclusion

The differential diagnosis of the biphasic mesenchimal uterine neoplasms could be difficult for the pathologist: after exclusion of the mixed mullerian tumors, the problems arise in the distinction of UTROSCTs versus leiomyoma with tubules. UTROSCTs were originally classified by Clement and Scully [1] into two groups. Type I were characterized by endometrial stromal tumors with sex cord-like elements (ESTSCLE: Group I tumors) while in type II tumors the sex cord-like elements predominated (UTROSCT: Group II). Although relatively few cases have been reported with long term follow-up, type I tumors showed a propensity to recur or metastatize, whereas type II followed a benign course. UTROSCT are placed in the miscellaneous category in the most recent World Health Organization classification [2], without any distinction between the different prognostic courses. Since the original description of UTROSCT several studies have attempted to further characterize this unusual group of uterine neoplasms with variable ultrastructural and immunohistochemical evidence supporting myogenic [3], epithelial [9,10] and sex cord differentiation [11-17].

In the present study we included an extended panel of antibodies inclusive of the identified markers of sex cord differentiation as reported in literature for the immunohistochemical analysis of UTROSCTs because the pathological features of the neoplasms described by us were reminiscent of ovarian sex cord tumor. The immunohistochemical profile of our cases were inconsistent with the previously described cases. They were negative for inhibin, CD99, CD10, calretinin and Melan A, weakly positive for Keratins in both sex cord areas and in stromal cells and immunoreactive for ER and PgR. In the following paragraphs we discuss the results of immunohistochemical results and compare these with previous studies in literature.

A review of the 47 cases of uterine tumors with sex cord differentiation reported in literature [11-14,16,17,19,20] shows inhibin expression in 17.6% (3/17) and 40% (12/30) of type I and type II tumors, respectively. These data are in contrast with the accurate diagnostic value of inhibin described in ovarian sex cord stromal tumors [9]. Therefore inhibin negativity should not preclude a diagnosis of UTROSCTs [25].

Of the previously reported UTROSCTs CD99 positivity was detected in five of five cases described by Baker et al [12] similar to Krishnamurthy et al [15] who found positivity in seven of seven UTROSCT. Oliva et al [20] reported CD99 positivity in four of seven cases and CD99 was detected in the single case report described by Motiwala et al [26], Oztekin et al [21] and Sutak et al [22]. Our neoplasms were not immunoreactive for CD99, although this antibody seems to be the most frequent marker of sex cord differentiation in UTROSCTs, more useful than inhibin.

CD99 expression tends to correlate with inhibin and it is typically confined to similar cell types in the individual tumors.

Calretinin is another marker useful and widely utilized for diagnosis of sex-cord tumors, and its value as marker of this group of neoplasms is well documented. In literature, to the best of our knowledge, description of calretinin immunoreactivity in UTROSCT is reported in 11 cases [13,18,22,23]: calretinin has been described positive in 67% (2/3) and 87.5% (7/8) type I and type II cases, respectively. The limited number of the reported cases don't give any conclusive information regarding the role of this marker in UTROSCTs diagnosis.

Keratins (high molecular weight AE1/3 and low molecular weight CAM 5.2) are usually used as a marker of epithelial differentiation, but it has been shown that some mesenchymal tumors may express this antigene, too. In the series of Oliva [20] and of Krishnamurthy [15], most of UTROSCTs stained for keratins (five of seven cases), a frequency similarly observed in other studies.

All of our cases exhibited nuclear immunoreactivity for estrogen receptors (ER) and progesterone receptors (PgR) as demonstrated in other studies. Seven examples of Clement and Scully's type II uterine tumors reported by Krishnamurthy et al [15] were found to be positive for ER and PgR in the gland-like formations. In the report of Irving et al [23] three of five cases of UTROSCTs showed ER immunoreactivity, although in two cases restricted to the sex cord elements, while four cases were PgR positive. In the case report diagnosed by Sutak et al as UTROSCT [22] staining for progesterone was negative. Our results are partially concordant with the data present in the literature, but we need further studies to determining the value of ER and PgR in the characterization of this polyphenotypic group of neoplasms.

H-caldesmon is useful to characterize the myoid differentiation but is usually negative in UTROSCTs although a component of smooth muscle can be seen in these neoplasms. Some authors suggest that cells with myoid appearance are an integral component of the tumor while others are uncertain whether these foci are entrapped smooth muscle [28].

Studies of Melan-A expression in UTROSCTs are limited to 17 cases [[15,17,24],29]. Of the previously reported type II – UTROSCTs 6 of 14 have been positive with Melan-A, whereas no positivity was demonstrated in 3 type I cases-ESTSCLE [15,17,23,24]. The positive findings of Melan-A supports a specialized gonadal stromal phenotype.

The literature review highlights that there aren't uniform or distinct immunohistological findings which characterize UTROSCT category. In the WHO classification UTROSCTs are classified as "sex cord – like tumors" in the "miscellaneous category". Nogales e Tavassoli think that these tumors are "histologically and immunohistochemically identical to ovarian steroid-producing cells, being strongly positive for alpha-inhibin, calretinin and CD99" [2], but the literature data contrast this affirmation. Consequently it may be problematic the diagnosis of a lesion which has histological features of UTROSCTs, without the immunohistochemical features, described before. In our cases, we preferred a descriptive nomenclature. The stroma of the lesions showed positivity for desmin, smooth muscle actin and caldesmon, confirming the smooth muscular differentiation of the stromal neoplastic component. In our lesions all markers of sex cord differentiation were absent, and so we preferred to classify the lesion as "leiomyoma with tubules". In according to diagnosis of Prof. Hildebrandt, Prof. Hendrickson, Prof. Kempson, we can conclude that a diagnosis based on histological features without any specification of the phenotype should be preferable in similar lesions when all the immunohistochemical markers for sex cord tumors are absent. The exact diagnosis is necessary for clinical in order to establish adequate prognostic criteria and optimal management of the lesion.

## Competing interests

The authors declare that they have no competing interests.

## Consent

Written informed consent was obtained from the patient for publication of this case report and any accompanying images. A copy of the written consent is available for review by the Editor-in-Chief of this journal.

## List of abbreviations

UTROSCT: Uterine tumors resembling ovarian sex cord tumor; ER: estrogen receptors; PgR: progesterone receptors

## Authors' contributions

AP was involved with the clinical management of the patient, TP and FP were involved in the design of the study and the preparation of the manuscript. All authors read an approved the final manuscript.
